# Patterns in Actions Against Physician Licenses Related to Substance Use and Psychological or Physical Impairment in the US From 2004 to 2020

**DOI:** 10.1001/jamahealthforum.2022.1163

**Published:** 2022-06-03

**Authors:** Lisa S. Rotenstein, Akanksha Dadlani, Jennifer Cleary, Srijan Sen, Anupam B. Jena, Douglas A. Mata

**Affiliations:** 1 Department of Medicine, Brigham and Women’s Hospital, Boston, Massachusetts; 2 Department of Medicine, Harvard Medical School, Boston, Massachusetts; 3 Northeast Ohio Medical University, Rootstown; 4 Department of Psychology, University of Michigan, Ann Arbor; 5 Department of Psychiatry, University of Michigan, Ann Arbor; 6 Department of Health Care Policy, Harvard Medical School, Boston, Massachusetts; 7 Department of Medicine, Massachusetts General Hospital, Boston; 8 Foundation Medicine, Cambridge, Massachusetts

## Abstract

This cross-sectional study examines the frequency of actions taken against physician licenses in the US because of substance use and psychological or physical impairment from 2004 to 2020.

## Introduction

Substance use, burnout, and depressive symptoms are prevalent among physicians,^1^ with potential consequences for patient outcomes and safety.^2^ Increased awareness of these issues has spurred efforts to support physicians.^3^ It is unclear to what extent these issues are reflected in actions taken by hospitals or licensing boards. The National Practitioner Databank (NPDB),^4^ a government database of adverse action reports and malpractice payments submitted by health care institutions and state licensing boards, facilitates examination of this topic. The aim of this study was to assess patterns in the actions against physician licenses owing to substance use and psychological health vs physical health.

## Methods

This cross-sectional study evaluated all physicians with actions against their licenses between 2004 and 2020 reported in the NPDB.^4^ We extracted data on physicians for whom the primary categorizations of license actions were owing to substance use, psychological health, or physical health impairments. The annual number of license actions in each category was normalized using data from the US Bureau of Labor Statistics.^5^ Characteristics of physicians with license actions were compared with those of the overall licensed US physician population.^6^ Differences among categorical and continuous variables were assessed using χ^2^ and Kruskal-Wallis tests, respectively. Analyses were performed using SAS OnDemand for Academics, and a 2-sided *P* < .05 was considered statistically significant. This study was exempt from institutional review board review on the basis of the Common Rule. We followed the STROBE reporting guideline.

## Results

Overall, 5032 actions against the licenses of US physicians between 2004 and 2020 were related to substance use (3841 [76.3%]), psychological impairment (577 [11.5%]), or physical impairment (614 [12.2%]). Despite a peak in 2011, actions related to substance use declined in frequency between 2004 and 2020 (slope, −0.21 [95% CI, −0.25 to −0.16] per calendar-year increase; *P* < .001) from 5.6 to 1.6 actions per 10 000 physicians (Figure). Actions related to psychological impairment slightly decreased between 2004 and 2020 (slope, −0.02 [95% CI, −0.03 to −0.008] per calendar-year increase; *P* = .004) from 0.8 to 0.2 actions per 10 000 physicians. Frequency of actions related to physical impairment also slightly decreased between 2004 and 2020 (slope, −0.01 [95% CI, −0.02 to −0.001] per calendar-year increase; *P* = .05) from 0.7 to 0.2 actions per 10 000 physicians.

**Figure.**
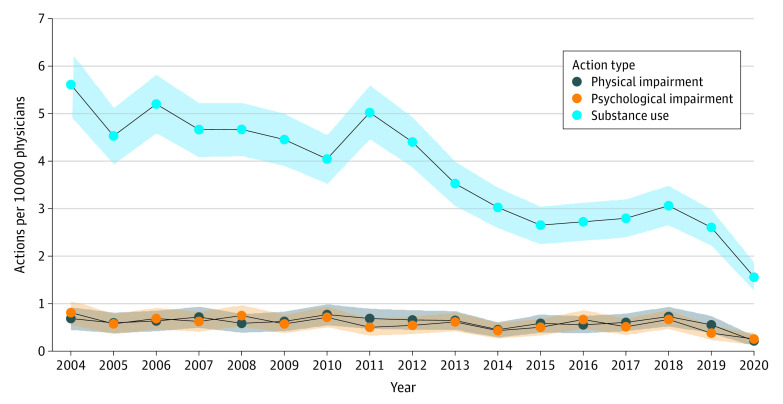
Frequencies of License Actions Related to Substance Use, Psychological Impairment, or Physical Impairment From 2004 to 2020 To facilitate comparisons over time, the yearly numbers of license actions in each category were normalized and expressed as the numbers of actions per 10 000 physicians using data on the US physician population from the US Bureau of Labor Statistics. The shaded areas represent the 95% CIs.

Compared with those with license actions related to physical impairment, physicians with license actions related to substance use or psychological impairment were more likely to have an indefinite rather than permanent penalty length (69.2% [2647 of 3826] and 80.6% [465 of 577], respectively, vs 58.1% [357 of 614]; *P* < .001), to have an emergency action taken against their license (12.0% [462 of 3841] and 20.6% [119 of 577], respectively, vs 8.0% [49 of 614]; *P* < .001) and to have a greater mean number of lifetime license actions (4.5 and 3.5, respectively, vs 2.5; *P* < .001) (Table).

**Table.** Adverse Actions Against Physician Licenses Between 2004 and 2020 Owing to Substance Use or Psychological or Physical Impairment

	No. (%)			*P* value for difference
	Substance use (n = 3841)	Psychological impairment (n = 577)	Physical impairment (n = 614)	
Age, y^a^				
<50	1723 (45.0)	184 (32.0)	107 (17.5)	<.001
≥50	2110 (55.0)	391 (68.0)	505 (82.5)	
US Census region^b^				
West	586 (15.5)	212 (37.8)	143 (23.7)	<.001
Midwest	1098 (29.1)	148 (26.4)	99 (16.4)	
South	1639 (43.5)	165 (29.4)	276 (45.7)	
Northeast	449 (11.9)	36 (6.4)	86 (14.2)	
Adverse action length^c^				
Indefinite penalty	2647 (69.2)	465 (80.6)	357 (58.1)	<.001
Permanent penalty	452 (11.8)	69 (12.0)	225 (36.6)	
Specified penalty	727 (19.0)	43 (7.5)	32 (5.2)	
Practitioners’ No. of licensure reports, mean (95% CI)	4.5 (4.4-4.6)	3.5 (3.3-3.8)	2.5 (2.3-2.7)	<.001
Adverse action classification				
License revocation	182 (4.7)	36 (6.2)	26 (4.2)	<.001
License probation	886 (23.1)	64 (11.1)	38 (6.2)	
License suspension	856 (22.3)	163 (28.2)	69 (11.2)	
Emergency license restriction, suspension, or revocation of clinical privileges	462 (12.0)	119 (20.6)	49 (8.0)	
Reprimand or censure	226 (5.9)	5 (0.9)	2 (0.3)	
Voluntary surrender, limitation, restriction, or agreement not to practice	457 (11.9)	97 (16.8)	238 (38.8)	
Limitation or restriction on license	226 (5.9)	28 (4.9)	42 (6.8)	
Suspension or revocation of clinical privileges	114 (3.0)	9 (1.6)	37 (6.0)	
Other license action	432 (11.2)	56 (9.7)	113 (18.4)	

^a^ Data were missing for 8 substance use, 2 psychological impairment, and 2 physical impairment actions.

^b^ Data were missing for 69 substance use, 16 psychological impairment, and 10 physical impairment actions.

^c^ Data were missing for 15 substance use actions.

## Discussion

This study of 5032 physician license actions in the US revealed that actions related to substance use have steadily declined during the past 17 years but remain markedly higher than those related to physical health. Actions related to psychological impairment were less common, but their frequency changed at a lower rate than those for substance use. Physicians with license actions related to substance use or psychological impairment were more likely to receive indefinite penalties and have an emergency action taken against their license, and they also had a greater number of license actions taken against them during their careers.

Limitations of this study include its retrospective nature, NPDB classification of actions into broad categories with limited information on the exact details of each license action, and that physician license actions represent only the most severe cases of impairment. Nevertheless, these findings suggest continued areas to improve mental health and support offerings for physicians, particularly interventions that may preclude the need for license actions in the first place.
